# Intestinal Dysbiosis in Rats: Interaction between Amoxicillin and Probiotics, a Histological and Immunohistochemical Evaluation

**DOI:** 10.3390/nu15051105

**Published:** 2023-02-23

**Authors:** Maria-Cătălina Matei-Lațiu, Adrian-Florin Gal, Vasile Rus, Victoria Buza, Cristian Martonos, Călin Lațiu, Laura-Cristina Ștefănuț

**Affiliations:** 1 Department of Animal Physiology, Faculty of Veterinary Medicine, University of Agricultural Sciences and Veterinary Medicine Cluj-Napoca, Calea Manastur No. 3-5, 400372 Cluj-Napoca, Romania; 2 Department of Histology, Faculty of Veterinary Medicine, University of Agricultural Sciences and Veterinary Medicine Cluj-Napoca, Calea Manastur No. 3-5, 400372 Cluj-Napoca, Romania; 3 Department of Anatomy, Faculty of Veterinary Medicine, University of Agricultural Sciences and Veterinary Medicine Cluj-Napoca, Calea Manastur No. 3-5, 400372 Cluj-Napoca, Romania; 4 School of Veterinary Medicine, Ross University, Basseterre P.O. Box 334, Saint Kitts and Nevis; 5 Faculty of Animal Sciences, University of Agricultural Sciences and Veterinary Medicine Cluj-Napoca, Calea Manastur No. 3-5, 400372 Cluj-Napoca, Romania

**Keywords:** dysmicrobism, antibiotics, TLR4, LBP, *Bacillus* spp.

## Abstract

Gastrointestinal microbiota can be easily altered by common treatments, such as antibiotic therapy. However, the dysmicrobism induced by such a treatment may be counteracted by the administration of different beneficial microbes, such as probiotics. Therefore, this study aimed to establish the interaction between intestinal microbiota, antibiotic therapy, and sporulated bacteria, correlated with the evolution of growth indices. Twenty-five Wistar rats, females, were divided into five groups. Amoxicillin and a probiotic combination of *Bacillus subtilis*, *Bacillus licheniformis,* and *Pediococcus acidilactici* were administered according to each group’s purpose. The conventional growth indices were calculated and histological and immunohistochemical assessments were realized from intestinal samples. The results of the conventional growth indices suggested a beneficial effect when the antibiotic therapy was accompanied by probiotics, while for the groups where the dysmicrobism was present, the values for feed conversion ratio were negative. These findings were supported by the microscopic aspects of the intestinal mucosa, which suggested a decreased absorption capacity due to significant morphological changes. Moreover, the immunohistochemical reaction of the inflammatory cells from intestinal lamina propria was intensely positive for the same affected groups. However, for the control group and the group with antibiotic and probiotic treatment, the immunopositivity was significantly decreased. Probiotics based on *bacillus* spores administered simultaneously with the antibiotic offered the best restoration of the gut microbiota, a fact suggested by the absence of intestinal lesions, a normal food conversion ratio, and low expression of TLR4 and LBP immunomarkers.

## 1. Introduction

The gastrointestinal microbiome is defined as a complex group of bacteria, archaebacteria, fungi, protozoa, and viruses that colonize the gastrointestinal tract of all mammals. Numerous studies in human and veterinary medicine have shown that the microbiome is involved in several vital physiological processes, such as maintaining the body’s homeostasis and metabolic processes, the health of the gastrointestinal epithelium, immune defence, and even neuro-behavioural development. The microbial genome greatly improves the activity and metabolic capacity of the host, thus becoming an active basis in the physiology of the colonized organism [1].

Changes in the gastrointestinal microbiome are associated with diseases in both animals and humans. These conditions often include inflammatory bowel disease, asthma, metabolic syndromes, obesity, cardiovascular disease, immune-mediated pathologies, or various neurodevelopmental disorders, such as autism spectrum disorders. Dysbiosis is defined as a condition of changes in the normal structure of the microbiome, associated with diseases or various pathologies that alter the microbial homeostasis of the host intestine. Dysbiosis is characterized by decreased numbers and bacterial diversity, changes in the intestine, but also systemic inflammation, and the altered metabolic relationship between the population of microorganisms and the host. One condition that affects the gut microbiome, regardless of species, is the administration of antibiotics [1].

The term probiotic is defined as a mixture of living microorganisms, which when consumed in adequate quantities provide the host with a health benefit [2]. The mechanisms by which the beneficial effects of probiotics are achieved are reduced intestinal permeability, increased mucin secretion by goblet cells, increased defensins that prevent colonization with pathogens, production of short-chain fatty acids, stimulation of IgA secretion, decreased pH, increase in the tolerance of immune cells to commensal microorganisms while for pathogens the tolerance is not diminished [1]. Probiotics can produce beneficial effects on the host without changing the permanent microbiome, this being possible through the transient colonization of the intestine. Thus, the administration of probiotics may protect intestinal homeostasis during antibiotic administration [1].

Conventional growth indices are often used to demonstrate the effect of certain substances that can serve as growth promoters or inhibitors. It is known that the alteration of the microbiological homeostasis, at the intestinal level, also affects intestinal absorption, which entails the modification of the growth and development indices. Both antibiotics and probiotics can influence the intestinal microbiome, indirectly leading to weight loss or gain [3].

Moreover, it is known that the gut microbial population directly influences the host’s immune defence capacity. Therefore, any alteration in the homeostasis of the intestinal microbiome directly affects the whole body, leading to a decrease in innate immunity [4]. Thus, any systemic treatment that reaches the intestinal level could affect the microbial populations at this level, leading to effects throughout the body. Antibiotic therapy and the administration of probiotics are two examples that change the composition of the intestinal microbiome, leading to effects such as intestinal inflammation, and increased frequency of allergic or autoimmune diseases [4]. Therefore, any change in the gut microbial population leads to an inflammatory syndrome. The initiation and perpetuation of intestinal inflammation may be the result of an exaggerated defence of the intestinal mucosa against endogenous bacteria in the lumen [5]. In this case, the inflammation is immune-mediated, based on a complex mechanism involving various cytokines and membrane receptors that is able to suggest the origin of inflammation, namely dysmicrobism. In this situation, the cells of the innate immune system are stimulated by structures expressed by microorganisms, structures known as pathogen-associated molecular patterns (PAMPs). These PAMPs interact with Toll-like receptors (TLRs), membrane or intracellular structures involved in the inflammatory cascade [6]. From the family of TLRs, the receptors activated in the context of dysmicrobism are represented by TLR2, TLR4, and TLR 5 [7].

Current evidence suggests that lipopolysaccharides (LPS), which are included in the PAMPs group and are present in the membrane of Gram-negative bacteria, can stimulate TLR4 activation. LPSs are among the most studied immunomodulatory components of bacteria that can induce systemic inflammation and even sepsis [6]. Moreover, LPSs are able, in vitro, to lead to immediate responses in enterocytes, which results in the production of cytokines and chemokines through various intercellular communication pathways, which use TLRs as mediators [5]. Stimulation of inflammatory cells through LPS includes a cascade of events involving the participation of several proteins such as LBP (LPS-binding protein), CD14, MD-2, and TLR4. LBP is a soluble protein that binds directly to LPS and facilitates its binding to CD14 [8]. In turn, CD14 facilitates the coupling of LBP with TLR4, modulating LPS recognition [6]. Thus, intestinal inflammation induced by dysmicrobism can be directly identified by analysing specific membrane markers such as LBP and TLR4.

The genetic information supports the direct involvement of TLRs, especially TLR4, in the production of intestinal inflammation and microbiota recognition. Thus, the increased epithelial expression of TLR4 is considered to be observable, especially in subjects with inflammatory bowel disease. However, insufficient data are available on the involvement of increased TLR4 expression in the maintenance of intestinal homeostasis. A study conducted in 2016 suggests that epithelial expression of TLR4 influences the composition of the microbiota and affects the defence properties of the epithelium. Thus, the innate immune system of the host can modulate the bacterial composition at the intestinal level and decrease the susceptibility of the host to intestinal inflammation [9].

The present study aimed to assess the interaction between intestinal microbiota, antibiotic therapy, and probiotics, correlated with the evolution of growth indices. To achieve this goal, histological and immunohistochemical assessments were performed, together with the evaluation of the conventional growth indices.

## 2. Materials and Methods

### 2.1. Study Design

The study included 25 white rats (*Rattus norvegicus*) of the Wistar breed, clinically healthy, females, weighing an average of 290.68 g (without significant differences between groups; *p* > 0.05 for Ordinary one-way ANOVA test). On the first day of the study, the subjects were randomly assigned to 5 homogeneous groups, 5 individuals each (Table 1).

The animals were hosted in the institutional biobase, in favourable conditions, adapted to their physiological needs. The environment was strictly controlled to avoid any external influence on the host. Experienced staff performed the care of the animals. The procedures were reduced to the minimum necessary to fulfil the purpose of the experiment.

### 2.2. Experimental Interventions

Amoxicillin capsules (250 mg), at a dose of 30 mg/kg body weight administered 2x/day, were used to induce intestinal dysmicrobism caused by antibiotic treatment. The antibiotic powder was suspended in water and administered daily by the gavage technique, for 14 days to subjects in groups 2 (ABX) and 4, and 7 days (the first 7 days of the study) to subjects in group 5 (ABX/PRB).

The used probiotic to counteract dysmicrobism was represented by a commercial formula, consisting of three bacterial species: *Bacillus subtilis* HU58 + *B. licheniformis* SL-307 + *Pediococcus acidilactici* Pedio 5051-4 × 10^9^ CFU, presented as capsules. The probiotic combination was selected because of the spore-forming bacteria in the composition. The content of one capsule was dissolved and homogenized in one millilitre of water, daily. The suspension was administered by gavage to groups 3 (PRB) and 4 (ABX + PRB) for 14 days and those in group 5 for 7 days (i.e., the last 7 days of the study). The dose administered was calculated to ensure the administration of 2 billion CFUs to each individual.

The daily body mass was determined for each individual before product administration. Moreover, food consumption was monitored daily for each experimental group. It must be mentioned that the present study did not determine the microbiota of the subjects, and this could be considered a limitation of the study.

### 2.3. Conventional Growth Indices Assessment

Feed conversion ratio (FCR), total body weight gain (TG), and specific growth rate (SGR) were calculated using the formulas presented in Table 2 [10].

### 2.4. Histological and Immunohistochemical Examination

At the end of the experiment, all 25 subjects were euthanized following the protocol recommended by applicable law and respecting the ethical guideline. The necropsy exam was performed immediately after the euthanasia. The anatomical identification of the intestinal segments and the sample harvesting were performed together with the previous step. Thus, the samples were represented by the proximal part of the duodenum, the distal part of the jejunum, and the middle segment of the colon. The histological slides obtained were analysed using an Olympus BX-41 microscope (Olympus, Japan). The microphotographs were obtained using a built-in Olympus U-TV0.35XC-2 T8 camera (Olympus, Tokyo, Japan).

#### 2.4.1. Histological Samples Preparation

All tissue samples were fixed in 10% buffered formalin for 5 days, followed by a progressive dehydration procedure using several alcoholic baths of different percentages (70%, 96%, and absolute ethanol). Afterwards, the clarification of the samples was realized using three repeated baths of 1-butanol (one hour each). The samples thus prepared were embedded in paraffin and sectioned using the Leica microtome (RM2125, Leica, Nussloch, Germany) into 5 µm sections. After displaying the sections on the slides, they were stained using Goldner’s trichrome technique [11].

#### 2.4.2. Tissue Sample Preparation for Immunohistochemical Assessment

The immunohistochemical technique was used to label and identify the receptors for the LBP and TLR4 markers. For their identification, 2 antibodies were used: anti-LBP (Lipopolysaccharide-Binding Protein (LBP) Antibody, code abx103461, Abbexa), and anti-TLR4 (TLR4 Polyclonal Antibody, code E-AB-64071, Elabscience). The samples were processed following the protocol presented for the histological assessment [11]. The samples were displayed on poly-L-lysine slides and then maintained for one hour at 60 °C and 24 h at 37 °C. The next step was to dewax the samples in xylene and hydration with alcohol series baths. The antigen retrieval of epitopes was performed by heat method for 16 min at 97 °C in a sodium citrate bath, pH 6.0 [12]. The blockage of endogenous peroxidase was performed with a 3% sodium peroxide solution (Elabscience 2-step plus Poly-HRP Anti Rabbit/Mouse IgG Detection System with DAB Solution). Afterwards, the incubation of the monoclonal antibodies for 24 h at 4 °C in a humid chamber was performed. The antibodies were used in diluted form; the dilution used (1/20 μg for LBP and 1:100 for TLR4, respectively) was selected according to the manufacturer’s instructions. The detection kit provided by Elabscience (2-step Plus Poly-HRP Anti Rabbit/Mouse IgG Detection System (with DAB Solution)) was used to develop the IHC (immunohistochemical) reaction as well as to detect immune markers. The colour reaction was obtained after the application of diaminobenzidine (DAB) for 60 s for LBP and 80 s for TLR4, respectively. The counter-staining was performed with Mayer Hematoxylin [11].

According to previously published protocols [7,13,14], immunopositivity for LBP and TLR 4 markers in the three intestinal segments was scored with a semi-quantitative method, assessing the extent and intensity of staining as follows: the extent of staining: no positive cells = 0, a few dispersed positive cells = 1, clusters of positive cells = 4, (almost) all cells positive = 7; the intensity of staining: faint = 0, present = 1, strong = 2. The obtained values for each assessment were added up to give a semi-quantitative staining score (ranging from 0 to 9) [7,13,14]. The evaluations were performed on five fields/animal/intestinal segments (40× magnification) in the representative sections by two pathologists. Additionally, the immunohistochemical aspect was categorized according to the intensity of the reaction on a scale from ± to +++, where ± represents a poor positive reaction, + a moderately positive reaction, ++ a clearly positive reaction, and +++ an intensely positive reaction. The immunohistochemical score was assigned by assessing the inflammatory cells in the intestinal mucosa, especially in the lamina propria.

### 2.5. Statistical Analysis

Sampled data were analysed in GraphPad Prism 8.0.1. ANOVA one-way test (α = 0.05) was used for both the comparison of the initial body weight of the rats to assess the uniformity of the groups and also to determine if the changes in terms of body weight of the rats from the five groups (G1 control group and G2 (ABX), G3 (PRB), G4 (ABX + PRB), and G5 (ABX/PRB) experimental groups) were statistically significant. Conventional growth indices were determined in MS Excel and were used to identify and quantify the effect of treatment on intestinal absorption. The score results for immunohistochemistry were expressed as median ± IQR (inter-quartile range). Differences between the scores of the same intestinal segment were assessed with the Kruskal–Wallis test, unpaired observations were analysed by the Mann–Whitney U-test, and *p* values ≤ 0.05 were considered statistically significant (i.e., G2 vs. G1, G3 vs. G1, G4 vs. G1, G5 vs. G1; G3 vs. G2, G4 vs. G2, G5 vs. G2).

## 3. Results

### 3.1. Conventional Growth Indices Assessment

ANOVA one-way test did not show statistically significant differences among the five groups (F = 0.4224; *p* = 0.7906; α = 0.05) in terms of body weight changes during the 14 days of the experiment.

**Figure 1:**
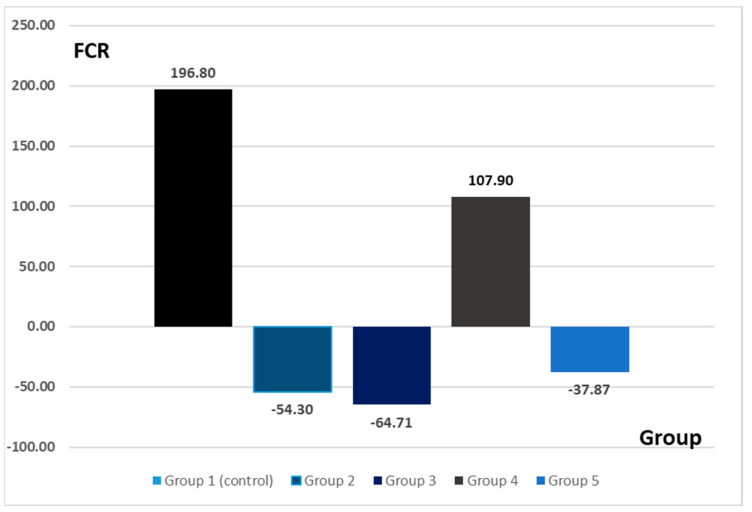
The feed conversion ratio for each experimental group.

The FCR values registered important differences between the experimental groups (Figure 1). The highest value for FCR was observed for G1 (control group). For the experimental groups where the alteration of the normal intestinal microflora was present, the values for FCR were negative in the groups G2 (ABX)(antibiotic), −54.30; G3 (PRB)(probiotic), −64.71; and G5 (7 days antibiotic + 7 days probiotic), −37.87. For G4, where the antibiotic-induced dysmicrobism was counteracted by the probiotic administration, the value for FCR was positive (107.9).

**Figure 2:**
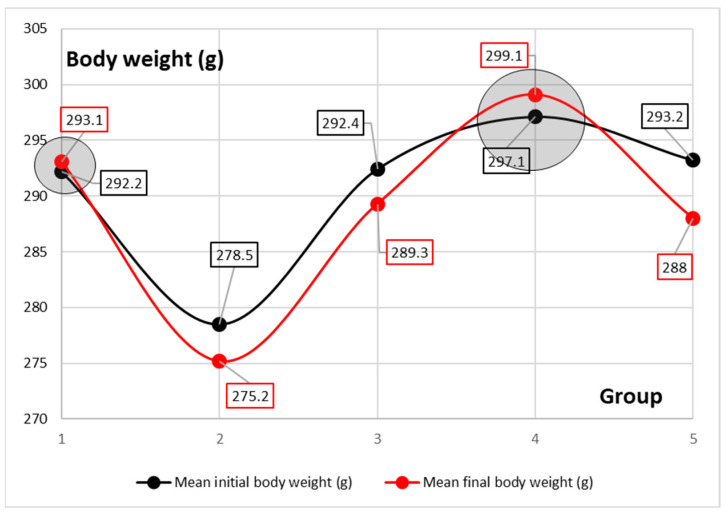
Mean differences between the initial body weight and final body weight for all the 5 groups.

Moreover, even if the quantity of feed consumed was similar in all the groups, for G2, G3, and G5, the values for total gain were negative (Table 3, Figure 2).

### 3.2. Histological and Immunohistochemical Examination

#### 3.2.1. Histological Features

**Figure 3:**
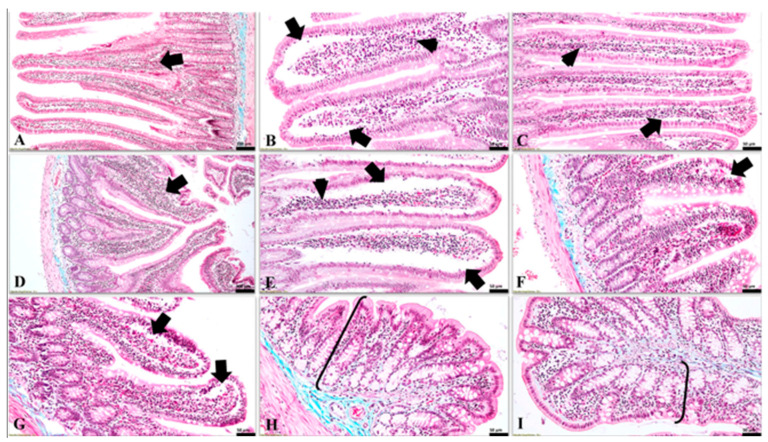
The microscopic features detected in the intestinal mucosa in all experimental groups (Goldner’s trichrome stain). (**A**) (G1, duodenum) normal aspect of the duodenal mucosa, and a moderate gut-associated lymphoid tissue (GALT) in lamina propria (arrow); (**B**) (G2 (ABX), duodenum) the presence of large subepithelial clefts (arrows), located mainly towards the apical pole of the duodenal villi along with abundant amount of GALT in the lamina propria (arrowhead); (**C**) (G3 (PRB), duodenum) noticeable subepithelial spaces (arrow) and moderate amounts of GALT in the lamina propria of the duodenum (arrowhead); (**D**) (G4 (ABX + PRB), duodenum) intestinal mucosa with a normal appearance and a prominent lymphoid tissue in the lamina propria of the duodenum (arrow); (**E**) (G5 (ABX/PRB), duodenum) large subepithelial clefts along the entire length of the duodenal villi (arrows) and an obvious mucosal-associated lymphoid tissue in the lamina propria (arrowhead); (**F**) (G2 (ABX), jejunum) intestinal villi in the jejunum with a discreet subepithelial space located mainly towards the apical poles and a moderate leukocytic infiltrate in the lamina propria; (**G**) (G3 (PRB), jejunum) jejunal villi with prominent subepithelial spaces towards their apical pole (arrow), in some cases these being visible up to the middle third of the villi; (**H**) (G2 (ABX), colon) and (**I**) (G3 (PRB), colon) absence of microscopic lesions in the mucosa (accolades) of the colon.

In the G1 (control group), as well as in G4 (i.e., the group treated simultaneously with antibiotic and probiotic), the changes in the intestine were absent or reduced (Figure 3). However, noticeable histological changes were identified in the duodenum and jejunum of the small intestine in groups 2, 3, and 5. The most important histological lesion was observed in duodenal mucosa (Figure 3A–E), which displayed subepithelial clefts with the preservation of the integrity and cohesion of the covering epithelium of the intestinal villi. The previously described lesion was found mainly at the apical pole of the intestinal villi. Similar but more discreet subepithelial clefts were detected in the villi of the jejunal mucosa (Figure 3F,G). In the colon, the histological changes were poorly represented or absent in all rat individuals from all groups (Figure 3H,I).

#### 3.2.2. Immunohistochemical Features

Regarding the immunolabeling of LBP and TLR4, the positive reaction was represented by different intensities of brown limited on the membrane of macrophages related to GALT (gut-associated lymphoid tissue) from the intestinal lamina propria. The intestinal segment with the highest intensity of the immunohistochemical reaction, for both LBP and TLR4 biomarkers, was detected in the duodenal mucosa followed by the jejunum. In the colon, the expression of the two markers is significantly lower compared to the previous segments.

**Figure 4:**
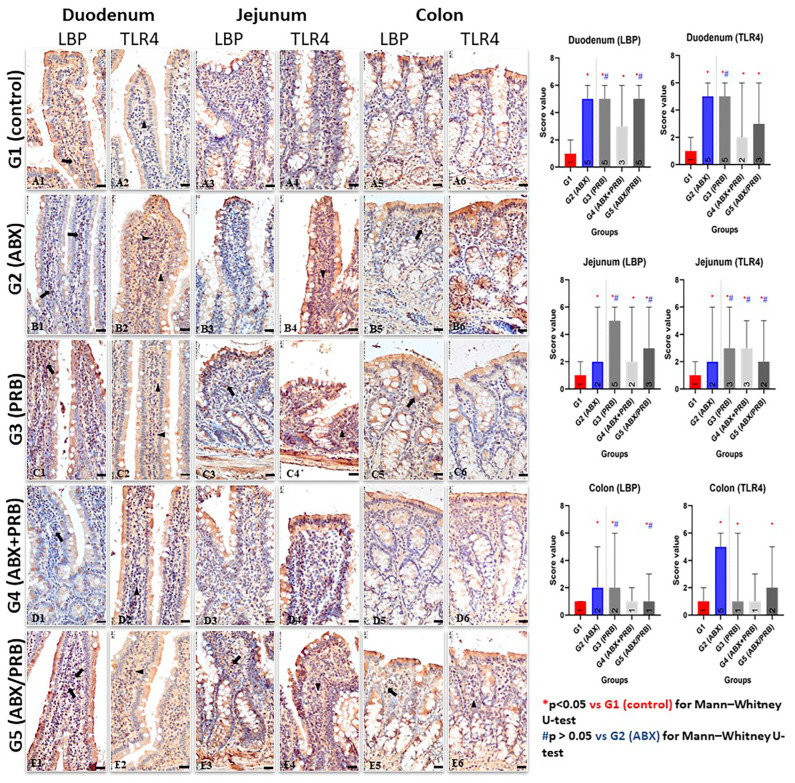
Expression of the LBP and TLR4 biomarkers in the duodenal (**A1**,**A2**,**B1**,**B2**,**C1**,**C2**,**D1**,**D2**,**E1**,**E2**), jejunal (**A3**,**A4**,**B3**,**B4**,**C3**,**C4**,**D3**,**D4**,**E3**,**E4**), and colonic (**A5**,**A6**,**B5**,**B6**,**C5**,**C6**,**D5**,**D6**,**E5**,**E6**) mucosa (anti-LBP IHC reaction and anti-TLR4 IHC reaction; Mayer’s hematoxylin counter-staining, 40× magnification, bar = 20 µm) and graphical representation of the staining scores with Mann–Whitney U-test results. (**A1**–**A6**) (G1 control group) faintly positive reaction of the macrophages, for both LBP (arrow) and TLR4 (arrowhead) immunomarkers. (**B1**) (G2 (ABX), duodenum) identification of numerous macrophages in the lamina propria exhibiting intense positive anti-LBP IHC-reaction (+++) (arrows); (**B2**) (G2 (ABX), duodenum) intensely positive anti-TLR4 reaction (+++) in macrophages related to the duodenal mucosa (arrowheads); (**B3**) (G2 (ABX), jejunum) intense anti-LBP immunolabeling (+++) of the macrophages from the jejunal mucosa; (**B4**) (G2 (ABX), jejunum): an intense membranary expression of TLR4 at GALT-associated macrophages (+++) from the lamina propria of the jejunum (arrowheads); (**B5**) (G2 (ABX), colon) clearly positive anti-LBP immunolabeling (++) of the macrophages related to the lamina propria of the colon (arrows); (**B6**) (G2 (ABX), colon) poorly positive immunoreactivity of TLR4 in the mucosa of the colon. (**C1**,**C2**) (G3 (PRB), duodenum) and (**C3**,**C4**) (G3 (PRB) jejunum) intense positive reaction in macrophages from lamina propria for both LBP (arrows) and TLR4 (arrowheads) immunomarkers; (**C5**,**C6**) (G3 (PRB) colon) clearly positive anti-LBP (++) (arrow) and anti-TLR4 immunolabeling of the macrophages related to the lamina propria of the colon. (**D1**) (G4 (ABX + PRB), duodenum) clearly immunolabeled GALT-associated macrophages (++) in the duodenal mucosa (arrows); (**D2**) (G4 (ABX + PRB), duodenum) clearly positive anti-TLR4 IHC reaction (++) in numerous macrophages in the duodenal mucosa (arrowhead); (**D3**,**D4**) (G4 (ABX + PRB), jejunum) clear positive anti-LBP and anti-TLR 4 immunolabeling (+) of the macrophages; (**D5**,**D6**) (G4 (ABX + PRB), colon) faintly positive reaction for both LPB and TLR4 immunomarkers. (**E1**) (G5 (ABX/PRB), duodenum) macrophages from duodenal mucosa that exhibit an intense immunoreaction (+++) for LBP immunomarker (arrows); (**E2**) (G5 (ABX/PRB), duodenum) intense immunopositivity of the macrophages for TLR3 immunomarker (arrowheads); (**E3**,**E4**) (G5 (ABX/PRB), jejunum) a prominent expression (+++) of the LBP biomarker (arrows) and TLR4 (arrowheads) in the mucosal-associated macrophages; (**E5**,**E6**) (G5 (ABX/PRB), colon): faintly positive for both immunomarkers (arrow and arrowhead).

For both immunomarkers (LBP and TLR4), Kruskal–Wallis statistical test showed significant differences between the scores of the groups in the same intestinal segments (*p* < 0.0001). At the duodenal level, Mann–Whitney U-test found differences between all the experimental groups and the control group for both LBP and TLR4 immunomarkers (*p* ≤ 0.0001). When the experimental groups with potential dysbiosis were compared with G2 (ABX), the results showed no significant differences for G3 (PRB) for LBP and TLR4, and G5 (ABX/PRB) for LBP (*p* > 0.05). At the jejunal level, Mann–Whitney U-test also suggested significant differences between experimental groups and the control group for both LBP and TLR4 markers (*p* < 0.05). At the comparison between G3 (PRB) and G5 (ABX/PRB) with G2 (ABX), no differences were found for both LBP and TLR (*p* > 0.05), while for the G4 (ABX + PRB), the results were insignificant only for TLR4. In the colon, the obtained scores suggested no significant differences in the comparison between G4 (ABX + PRB) and G1 (control) (*p* > 0.05), while for the other groups (G2 (ABX), G3 (PRB), and G5 (ABX/PRB), Mann–Whitney U-test demonstrated differences between the obtained scores (*p* < 0.0001). For LBP, no differences between G3 (PRB) vs. G2 (ABX) and G5 (ABX/PRB) vs. G2 (ABX) were observed (*p* > 0.05) (Table S1, Figure 4 and Figure S1).

Concerning the comparative expression of the two assessed biomarkers, the immunohistochemical reaction was comparable, a fact that suggests a direct relationship between LBP and TLR4 in each analysed intestinal segment. Regarding the site where the two markers are expressed, they were identified in GALT, mainly in the macrophages of the lamina propria.

At the duodenal level, where the immunopositivity score was highest compared to the other two intestinal segments, the clear expression of the LBP biomarker in the membrane of macrophages from the lamina propria was noted (Figure 4B1,C1,E1). Among the groups analysed, an intensely LBP-positive (+++) reaction was observed in groups with dysmicrobism, namely G2 (Figure 4B1), G3 (Figure 4C1), and G5 (Figure 4E1). For the control group (G1), in which the dysmicrobism was not induced, the immunohistochemical reaction was weakly positive (±) (Figure 4A1). Regarding the immunoreactivity of the duodenal mucosa for the TLR4 biomarker, in the case of G1 it was of low intensity (±) (Figure 4A2), whereas in the macrophages from the lamina propria of the rat individuals from G2 (Figure 4B2) and G3 (Figure 4C2) an intensely positive reaction was observed (+++). Similarly, a clearly TLR4-positive (++) reaction was noticed in the duodenal samples from G4 (Figure 4D2) and G5 (Figure 4E2).

The expression of the LBP biomarker at the level of the jejunal mucosa registered different intensities depending on the analysed group. Thus, for G1, the immunoreaction had a low intensity (±) (Figure 4A3). However, in G2 the GALT-associated macrophages were intensely LBP-positive (+++) (Figure 4B3), whereas in G3 (Figure 4C3), G4 (Figure 4D3), and G5 (Figure 4E3) the expression of the aforementioned biomarker was clear (++). As regards the expression of TLR4 biomarker in the jejunal mucosa, an intensely positive reaction was observed in macrophages from the lamina propria of the rat individuals belonging to G2 (+++) (Figure 4B4) vs. G3, G4, and G5 that displayed a slightly lower reaction (++). Comparatively, in the jejunal mucosa of the rats belonging to G1, the anti-TLR4 immunohistochemical reaction was poor (±).

Immunoreactivity of the mucosa in the colon recorded lower scores compared to the small intestine. Thus, for the LBP biomarker, the only group in which the reaction was clearly positive (++) was recorded in G2 (Figure 4B5) vs. G3, G4, and G5 in which the IHC reaction was moderately positive (+), whereas in the control group, the LBP expression was poorly positive (±). Regarding the expression of TLR4 biomarker in the lamina propria of the colon, the tissue samples from the G1 showed a poorly positive reaction (±) vs. G2, G3, G4, and G5 that showed a moderate (+) reaction (Figure 4E6).

## 4. Discussion

The highest value of the feed conversion ratio was recorded in G1 (i.e., the control group). Given that the experimental interventions on this group were limited to the administration of a placebo product without any influence on the digestive system, this effect can be considered as expected. For G2 (14-day amoxicillin administration) and G3 (14-day probiotic administration), the value of the feed conversion ratio was negative. Amoxicillin has been used due to its increased intestinal bioavailability [15]. The negative values can be explained due to the ability of the two administered products to modify the intestinal microbiome and implicitly influence the intestinal absorption rate. Thus, although the animals consumed a normal amount of food, similarly to the control group, they lost weight. This effect can be directly attributed to the experimental intervention. Regarding G4 (antibiotic and probiotic ingested concomitantly for 14 days), the feed conversion ratio recorded positive values compared to the control group. The amount of food consumed was also within physiological parameters, being comparable to groups G1, G2, G3, and G5. Thus, the positive value recorded for FCR in this group is the result of the concomitant administration of the two treatments. Regarding G5 (antibiotic 7 days followed by probiotic 7 days), the FCR value was negative. In the context of a quantity of food consumed approximately equal to the other groups, it can be considered that the probiotic administered after the cessation of the antibiotic treatment does not have the expected effect.

The modification of the intestinal microbiome can lead to digestive problems that can be observed through conventional growth indices [16]. A study conducted in 2009 states that treatment with probiotic bacteria significantly affected the evolution of the body weight of rats, with the intervention group showing a decrease in body weight compared to the control group [3]. The same observation is valid in the present study, where the dynamics of body mass in groups 2, 3, and 5 showed a negative direction compared to groups 1 and 4. In the case of the three groups with negative FCR (G2, G3, and G5), the experimental interventions (antibiotic/probiotic administration) induced intestinal dysmicrobism which implicitly affected the ability to metabolize food. If the results on the administration of antibiotics are clear, then in terms of the administration of probiotics, the available data are contradictory. Thus, the results of another study suggest that the administration of *B. subtilis* and *B. coagulans* in rats led to an improvement in the rate of feed conversion, leading to weight gain despite a low feed intake [17]. The administration of *L. acidophilus* also led to an improvement in the rate of feed conversion in rats compared to the control group [18].

The values of the conventional growth indices obtained for the groups where the dysbiosis was present (i.e., G2—antibiotic, G3—probiotic, G5—antibiotic 7 days + probiotic 7 days) can be linked with the morphological aspects observed at the histological assessment. Negative values of the growth indices suggest weight loss despite adequate food intake. That phenomenon may be explained by poor duodenal absorption. The histological aspects observed at this level support this hypothesis. The administration of probiotics is considered to be able to influence the absorption of nutrients in the intestine. The mechanism underlying this hypothesis is the stimulation of the growth of beneficial bacterial populations that would maintain the health of the intestinal system [17]. On the other hand, absorption in the small intestine is affected by the state of dysmicrobism [19], [16]. The fact that the administration of probiotics could induce transient dysmicrobism could explain both the negative values of the food conversion ratio obtained for G3 (by affecting the absorption capacity) and the histological aspects at the duodenal level.

Another characteristic attributed to probiotics is the protection of the intestinal mucosa against inflammation, especially in the colon, one of the mechanisms being the increase in the number and secretory activity of goblet cells [16]. However, the expression of certain receptors involved in inflammation (such as TLR4) is thought to influence cell differentiation and lead to changes in the number of goblet cells in the gut. At present, it is considered that the change in the number of goblet cells is present in the small intestine but not in the colon [9]. In the present study, when the groups are compared, the number of goblet cells does not register a significant change. Other studies also show that the administration of probiotics based on *bacillus* spores has a beneficial effect on the colonic mucosa during inflammation [20]. It is considered that some bacteria from the *Bacillus* genus present a good probiotic potential due to their capacity to form spores and, therefore, to enhance their viability in difficult conditions, such as low pH in the GIT [2,21,22,23] or the presence of digestive enzymes [24,25].

On the other hand, it is known that probiotics can have a trophic effect on the intestine. Thus, the use of probiotics can improve the proliferation rate of enterocytes, leading to the strengthening of the intestinal barrier and thus to an increased defence capacity against pathogens. In contrast, pathogenic bacteria can reduce the rate of enterocyte proliferation, leading to mucosal atrophy and decreased immune defence capacity in the intestine. The administration of probiotics in the case of dysmicrobism, when the intestinal mucosa is affected, involves the annihilation of the hypoproliferative status of the cells, including improving the intestinal absorption capacity [26]. The histological aspects observed in this study are indirectly correlated with this information. Starting from the premise that intestinal dysmicrobism directly affects the intestinal mucosa and, implicitly, its absorption capacity, the present study found in subjects from the experimental groups (in which dysmicrobism was induced) the presence of subepithelial spaces following detachment of the covering epithelium from the underlying basement membrane, which thus remains bare. However, in the groups where the dysmicrobism was absent or poorly represented (i.e., G1 and G4), the histological feature presented above is directly proportional to the intensity of the dysmicrobism.

Nevertheless, bacteria or their products may penetrate the epithelial barrier. This is due either to mucosal lesions or to specific mechanisms that have the ultimate goal of immune stimulation of the intestinal mucosa. Thus, inflammatory products will interact with the apical portion of enterocytes, inducing responses in these cells. These cellular reactions are represented by the production of various mediators of inflammation, with the role of activating the immune response in the mucosa [5]. Although these mechanisms occur at the molecular level, their manifestation may be histologically visible. Hence, starting from the premise that intestinal dysmicrobism will activate the inflammatory cascade and cause changes in the intestine, the denudation of the basement membrane may be one of the consequences. Moreover, the above-mentioned feature, along with the presence of the subepithelial clefts, was detected at the duodenal level in the groups in which the dysmicrobism was representative (i.e., the rats from G2, G3, and G5).

In the present study, the two membrane markers associated with intestinal dysmicrobism (LBP and TLR4) were analysed immunohistochemically. In all groups in which dysmicrobism was present, the gut-associated macrophages from the lamina propria of the small intestine expressed high levels of LBP and TLR4 biomarkers. It is known that amoxicillin can induce dysmicrobism by altering the intestinal microbial composition, leading to marked intestinal inflammation [4]. Indeed, in group 2, where dysmicrobism was induced by amoxicillin administration, an intensely positive reaction was observed in the duodenal and jejunal levels, and a moderate reaction in the colon. In 2020, Graversen et al. demonstrated that amoxicillin administration affects the composition of the intestinal microbiome, promoting intestinal inflammation and regulating the immune response [4]. In fact, the immune response is modulated by inflammation present in the intestine, inflammation mediated by LPS and TLR4. Thus, it can be considered that probiotics can modulate the immune response by the same mechanism. Even though probiotic bacteria are considered beneficial bacteria to the host, their administration to healthy individuals can induce a transient state of dysmicrobism, which leads to intestinal inflammation (as observed in our study). This possible explanation is supported by the immunohistochemical observations identified in G3, where the administration of the probiotic to healthy animals produced a marked intestinal inflammation, especially in the small intestine. Furthermore, similar positive results for both immunomarkers were detected in G5, i.e., rat individuals that underwent administration of amoxicillin for one week followed by the administration of the probiotic (7 days). The semiquantitative staining scores also suggest that probiotics alone produce similar effects with the antibiotic administered in G2 (ABX). As explained above, both treatments were able to produce a change in the microbial community at the intestinal level, a fact that may explain the presence of comparative levels of inflammation in both cases. Thus, the inflammatory state produced by the probiotic administration may be related to their capacity to modulate the immune system in a nonspecific pathway [5].

In rats from G4, the morphological aspect was different. The intensity of the immunohistochemical reaction for both LBP and TLR4 biomarkers was weak or moderately positive for all intestinal segments assessed. This can be explained by the antagonism between probiotics and antibiotics [27,28]. As a comparison, other reports suggested beneficial effects in humans and in experiments performed on mice, in which the administration of probiotics has led to a balanced immune response [4,29]. In the present study, the degree of dysmicrobism was directly influenced by the counteracting effect of amoxicillin by the probiotic. Moreover, several studies suggest that sporulated bacteria from the *Bacillus* genus may present antibiotic resistance [30,31,32], thus should not be killed in case of co-administration. Consequently, the direct effect on the intestine was diminished, the inflammation being low in intensity. The efficacy of probiotics in various pathologies associated with intestinal dysmicrobism is extensively studied. Regarding intestinal inflammation, the results presented in this study are similar to the results of other researchers who demonstrated the beneficial effect of probiotic treatment in antibiotic-induced dysmicrobism [28,33,34]. It is known that the administration of certain antibiotics (e.g., vancomycin) can induce inflammation in the colon. A study conducted in 2003 showed that the administration of probiotics in antibiotic-induced colitis significantly reduced the lesion score [28]. In our study, amoxicillin-induced inflammation in the colon was low in intensity, which is why the assessment of the countering effect of probiotics in antibiotic-induced colitis is of low relevance.

The degree of inflammation described in subjects from G4 (i.e., rats free of any experimental intervention) was similar to the one observed in the control group, in which the inflammation in the three segments evaluated was classified as weakly positive. The presence of TLR4-positive cells in the gut of healthy individuals has been previously reported as a normal feature [5]. The presence of LPS in the lumen is well tolerated when the intestinal mucosa does not show pathological changes. This can be explained by limiting the activity of TLR4 receptors involved in the recognition of LPS. On the other hand, acute lesions in the intestinal mucosa will lead to the activation of TLR4-positive macrophages, cells that also have active CD14 receptors, which favours the binding of inflammatory complexes to specific receptors and thus the activation of the inflammatory cascade in the intestine [5].

## 5. Conclusions

The gut microbiota, an intricate population of microorganisms, is significantly involved in intestinal homeostasis, but it may exert an essential influence on the host in various gastrointestinal disorders. Several experimental models were developed over time in an effort to find the best way to study “the dysbiotic microbiota”. The experimental model that utilizes amoxicillin is suitable for the study of intestinal microbiota since it was able to produce severe intestinal dysmicrobism, as demonstrated by the expression of TLR4 and LBP membrane biomarkers in the gut-associated lymphoid tissue, mainly in macrophages. In our study, conventional growth rates were directly influenced by the state of dysbiosis. Negative values of food conversion ratio were associated with dysbiosis, which triggered some intestinal lesions in the small intestine (e.g., intestinal villi with subepithelial clefts along with a denudated basement membrane) that could be responsible for the poor intestinal absorption. Probiotics based on *bacillus* spores administered simultaneously with the antibiotic offered the best restoration of the gut microbiota, a fact suggested by the absence of intestinal lesions, a normal food conversion ratio, and low expression of TLR4 and LBP immunomarkers. However, the symbiotic relationship between the host and the intestinal microbiota has deleterious effects following the administration of the probiotic in dysbiotic-free individuals. Similarly, the intestinal microbiota was not restored in rats in which the probiotic was administered after 7 days of amoxicillin. The understanding of the gut microbiota microenvironment, including its composition and management in various intestinal or non-intestinal disorders, might be useful to investigate various microbiome modulators capable of restoring the symbiotic host–microbiota relationship to provide its full beneficial properties.

## Figures and Tables

**Table 1:** Biologic material repartition in study groups.

Group	Animals	Average Body Weight at the Beginning of the Study (g)	Group Destination
1	5 ♀	292.20 ± 32.72 ^1^	Control—vector administration—14 days
2	5 ♀	278.50 ± 25.83 ^1^	Antibiotic administration—14 days
3	5 ♀	292.40 ± 26.22 ^1^	Probiotic administration—14 days
4	5 ♀	297.10 ± 9.18 ^1^	Antibiotic and probiotic administration—14 days
5	5 ♀	293.20 ± 25.53 ^1^	Antibiotic administration (7 days) followed by probiotic administration (7 days)

^1^ No significant differences between groups for Ordinary one-way ANOVA test; *p* > 0.05.

**Table 2:** Formulas for conventional growth indices.

Feed Conversion Ratio	Total Gain	Specific Growth Rate
FCR = Q_h_/TG	TG = Wg_f_ − Wg_i_	SGR = 100 × [ln(Wg_f_) − ln(Wg_s_)]/T

Q_h_—the total amount of food consumed for 14 days in each group; TG—total body weight gain of the group; Wgf—final body weight of the group; Wgi—initial body weight of the group; T—the total number of days of the experiment.

**Table 3:** Values for all the growth conventional indices.

Group	Feed Consumed	Total Gain	FCR/Group	SGR
G1	885.8	4.50	196.80	0.021967
G2 (ABX)	896	−16.50	−54.30	−0.08514
G3 (PRB)	1003	−15.50	−64.71	−0.07613
G4 (ABX + PRB)	1079	10.00	107.90	0.047923
G5 (ABX/PRB)	984.5	−26.00	−37.87	−0.12782

FCR—feed conversion ratio; SGR—specific growth rate.

## Data Availability

Not applicable.

## Supplementary Materials

The following are available online at https://www.mdpi.com/article/10.3390/nu15051105/s1, Table S1: Semi-quantitative staining scores [median (IQR), range 0–9] for LBP and TLR 4 markers in the 3 intestinal segments. Figure S1: Graphical representation of the staining scores, represented as mean ± SEM and Mann–Whitney U-test results.
