# Supplementary material for: Intestinal Dysbiosis in Rats: Interaction between Amoxicillin and Probiotics, a Histological and Immunohistochemical Evaluation
Source: Nutrients. 2023 Feb 23;15(5):1105. doi: 10.3390/nu15051105 (PMC10004829; doi:10.3390/nu15051105)
Supplement: Supplementary file 1 [file nutrients-15-01105-s001.zip › nutrients-2185242-supplementary.pdf]

Supplementary file

Table S1. Semi-quantitative staining scores [median (IQR), range 0–9] for LBP and TLR 4 markers in the 3 intestinal segments.

Group	Intestinal segment	LBP	TLR4
G1	Duodenum	1 (0 – 2)	1 (0 – 2)
	Jejunum	1 (0 – 2)	1 (0 – 2)
	Colon	1 (0 – 1)	1 (0 – 2)
G2 (ABX)	Duodenum	5 (0 – 6)*	5 (1 – 6)*
	Jejunum	2 (1 – 6)*	2 (1 – 6)*
	Colon	2 (0 – 5)*	5 (2 – 6)*
G3 (PRB)	Duodenum	5 (1 – 5) *#	5 (1 – 6)*#
	Jejunum	5 (1 – 6)*#	3 (1 – 6)*#
	Colon	2 (0 – 6)*#	1 (1 – 6)*
G4 (ABX+PRB)	Duodenum	3 (0 – 6) *	2 (0 – 6)*
	Jejunum	2 (1 – 6)*	3 (1 – 5)*#
	Colon	1 (0 – 2)	1 (0 – 3)
G5 (ABX/PRB)	Duodenum	5 (1 – 5) *#	3 (0 – 6)*
	Jejunum	3 (1 – 6)*#	2 (1 – 5)*#
	Colon	1 (0 – 3)*#	2 (1 – 5)*

*p<0.05 vs G1 (control) for Mann–Whitney U-test

#p > 0.05 vs G2 (ABX) for Mann–Whitney U-test

Figure S1. Graphical representation of the staining scores, represented as mean ± SEM and Mann–Whitney U-test results

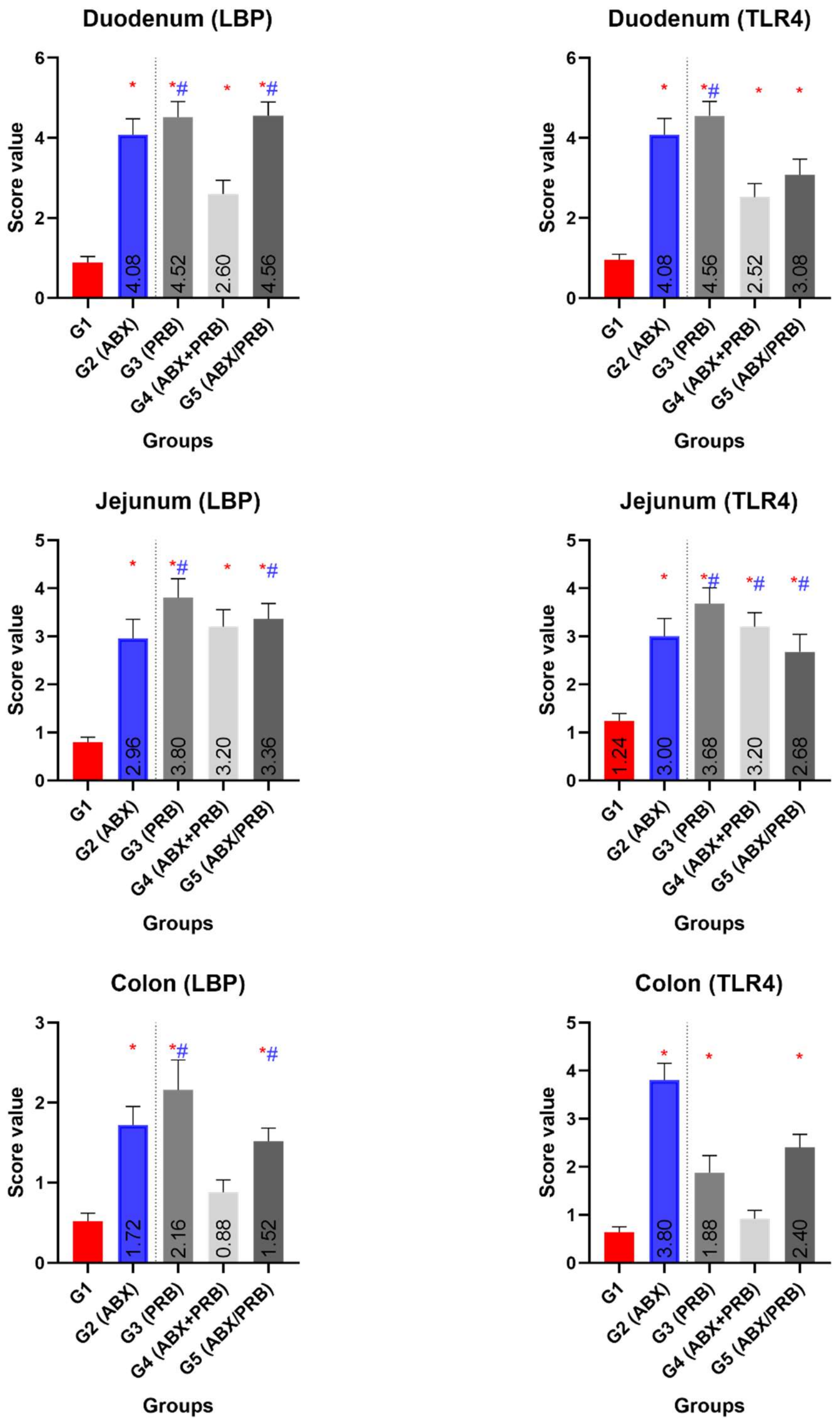

*p<0.05 vs G1 (control) for Mann–Whitney U-test
#p > 0.05 vs G2 (ABX) for Mann–Whitney U-test
